# Human and Small Animal Detection Using Multiple Millimeter-Wave Radars and Data Fusion: Enabling Safe Applications

**DOI:** 10.3390/s24061901

**Published:** 2024-03-16

**Authors:** Ana Beatriz Rodrigues Costa De Mattos, Glauber Brante, Guilherme L. Moritz, Richard Demo Souza

**Affiliations:** 1 Department of Electrical and Electronics Engineering, Federal University of Santa Catarina (UFSC), Florianopolis 88040-900, Brazil; ana.beatriz.mattos@posgrad.ufsc.br; 2 Academic Department of Electrotechnics, Federal University of Technology—Paraná (UTFPR), Curitiba 80230-901, Brazil; gbrante@utfpr.edu.br (G.B.); moritz@utfpr.edu.br (G.L.M.)

**Keywords:** millimeter-wave, data fusion, multiple radars, small animals, human tracking, wireless power transfer

## Abstract

Millimeter-wave (mmWave) radars attain high resolution without compromising privacy while being unaffected by environmental factors such as rain, dust, and fog. This study explores the challenges of using mmWave radars for the simultaneous detection of people and small animals, a critical concern in applications like indoor wireless energy transfer systems. This work proposes innovative methodologies for enhancing detection accuracy and overcoming the inherent difficulties posed by differences in target size and volume. In particular, we explore two distinct positioning scenarios that involve up to four mmWave radars in an indoor environment to detect and track both humans and small animals. We compare the outcomes achieved through the implementation of three distinct data-fusion methods. It was shown that using a single radar without the application of a tracking algorithm resulted in a sensitivity of 46.1%. However, this sensitivity significantly increased to 97.10% upon utilizing four radars using with the optimal fusion method and tracking. This improvement highlights the effectiveness of employing multiple radars together with data fusion techniques, significantly enhancing sensitivity and reliability in target detection.

## 1. Introduction

The Internet revolution has significantly altered the way people access, search, and disseminate information through the interconnection of devices around the world [1]. The emergence of the Internet of Things (IoT) is creating a bridge between the virtual and the real world, necessitating scalable mobile networks to accommodate the demands of an estimated dozen billion connected devices [2]. Moreover, the processing capabilities must evolve to handle the vast amount of information generated by these digital entities [2]. Projections anticipate a staggering 75 billion connected devices by the end of 2025 [3]. Using advanced sensors, as mmWave radars, embedded within everyday objects, the IoT empowers intelligent data-driven decision-making in various industries [1]. MmWave technology is instrumental in advancing IoT applications across various domains, including smart homes, wearables, and smart cities, by enhancing, e.g., intelligent surveillance, automated transportation, and security measures with unparalleled accuracy and efficiency [4]. This evolution underscores the growing importance of integrating sensing technologies, such as mmWave radars, into the IoT infrastructure to realize its full potential in enabling smart environments and applications [5].

With the advances in IoT technologies, the demand for high-precision, secure, and private location monitoring has increased significantly. Location monitoring and movement tracking are of critical importance in various scenarios, such as smart homes, indoor navigation, security surveillance, disaster management, and smart healthcare [6]. Among the array of sensors used to detect people, gestures, and objects, cameras and radars are known for their cost effectiveness while maintaining commendable precision levels compared to other sensor technologies [7]. Current research on detection and tracking employs various sensing approaches and algorithms, such as passive infrared sensors (PIR) [8,9], light detection and ranging (LIDAR) [10,11], and digital cameras [12,13,14]. However, each of these technologies faces challenges related to accuracy, privacy, and environmental robustness [15,16].

Millimeter-wave (mmWave) radars employ short-length electromagnetic waves, resulting in high precision. Unlike technologies such as cameras and LIDAR, radar measurements are less affected by environmental factors such as rain, fog, and dust [15], while also preserving privacy. Additionally, radar can achieve high-range and high-speed object detection [15]. A prominent example of commercial radar systems is the IWR6843 mmWave sensor from Texas Instruments (TI) [17]. These sensors produce point clouds: three-dimensional datasets that convey object positions in three axes, Doppler data, angles for each point, and other relevant information, providing comprehensive environmental data [18]. A common use case of mmWave radar is in the detection and tracking of humans.

However, the literature is scarce on methods capable of detecting and tracking humans and animals in the same environment. By identifying the presence of animals, sensors facilitate early alerts to drivers, machine operators, security personnel, and activation of safety measures, thus reducing the risks of potential incidents [19]. Furthermore, the detection of animals through sensors promotes safe cohabitation in shared environments [19]. Taking into account that there are around a billion pets worldwide (https://www.healthforanimals.org/, accessed on 25 February 2024), the ability to detect and track humans and small animals may result in many novel applications.

### 1.1. Related Work

Several works have explored detecting or tracking people using mmWave radar [7,16,18,20,21]. The work in [7] presents an identification system named mID, utilizing mmWave radar technology. Meanwhile, the authors in [21] introduce an extended object-tracking Kalman filter capable of estimating the position, shape, and extension of the subjects. It integrates a novel deep-learning classifier designed specifically for efficient feature extraction and rapid inference from radar point clouds. Additionally, the work in [22] implements an mmWave radar-based multi-person tracking system utilizing a single radar.

Moving forward, combining sensors through data fusion has emerged as a promising approach to gaining additional information in various applications [23]. The data fusion process involves multiple stages, including detection, association, correlation, estimation, and combination [23]. It encompasses the fusion of data from similar or dissimilar sensors. For instance, in a multi-sensor system comprising identical sensors, a target detected by several sensors provides estimation states to the fusion center for target tracking [23]. Additionally, the work in [24] showcases the effective fusion of information from multiple radars, resulting in improved area coverage, probability of detection, localization, and tracking performance.

In this line, multi-radar tracking can be seen as a way of obtaining a view of an object from two or more angles simultaneously [25]. According to [25], the use of multiple radars has some advantages and disadvantages. Some of the advantages are the better resolution in the presence of noise, detection uncertainties, and more reliable tracking data [25]. The disadvantages would be the constant communication between the radar platforms and the increased amount of data processing [25]. Various radar fusion techniques are presented in [23], employing a coefficient calculation method based on the trace of the error covariance matrix. The use of the strong tracking filter (STF) is introduced in the estimation of the target state, demonstrating superior performance compared to conventional or extended Kalman filters. This integration improves the overall target tracking performance.

In [26], a simulation utilizing the fusion of multiple radars suggests that employing two radars results in a higher detection probability and higher precision compared to a single radar. Furthermore, [27] introduces a multi-radar calibration method by tracking pedestrian trajectories. The fusion of multiple radars has shown utility in estimating human posture. In [28], two mmWave radars were strategically placed: one detecting (x,y) data and the other capturing (x,z) data to collect reflection points. A neural network was used for data fusion. In [29], an algorithm called Pontilism was introduced for a system of multiple radars. This algorithm addresses specular reflections, sparsity, and noise in radar point clouds, enhancing radar perception with 3D bounding boxes. The study demonstrated that the use of multiple radars resulted in a reduced error compared to using a single radar.

There are some works in the literature that use point clouds generated from multiple radars to specifically detect and track people, as in [16,18,30,31], where different radar positioning scenarios were proposed. For example, the work in [30] introduced a human tracking system based on mmWave radar, employing two radars placed along the walls of a room. This setup enabled the detection of moving humans by sparse point clouds. Similarly, the authors of [16] investigated the use of two mmWave radar sensors for accurate people detection and tracking. However, their radar positioning differed, with the radars located at the corners of the room. Furthermore, a real-time system framework is proposed in [31] to merge radar signals to track human position and body status. Unlike previous studies, the authors utilized a configuration involving three radars, one placed on the ceiling and the other two on the walls, ensuring precise tracking accuracy.

In the pioneering study using point clouds from multiple mmWave radars presented in [18], a software framework capable of communicating with multiple radars and applying a customized data-processing chain is introduced. These radars were placed on the walls of a room. The conclusion shows that the proposed system achieves over 90% sensitivity in indoor human detection. In particular, using a two-radar configuration significantly improves precision from 46.9% to an impressive 98.6%. However, in this case, the sensitivity decreased from 96.4% to 90.4%. Depending on the application, as those concerned with security or safety aspects, a reduction in sensitivity may be unacceptable. Moreover, the authors discuss the potential interference among multiple radars, showing that the probability of interference when using four radars is less than 1%. However, such a probability increases considerably with more than ten radars, which would then require explicit synchronization between radars or an interference-detection algorithm.

Unlike human detection, the detection of animals presents a distinct challenge, given the variations in size and shape. The work presented in [32] explores in-phase and quadrature (IQ) radar data on humans and animals, focusing on the extraction of radar-data-distinguishing features to classify animals versus humans based on micro-Doppler signatures. Additionally, in [33], the use of radar micro-Doppler signatures for the automatic contactless identification of lameness is presented, showing preliminary results for dairy cows, sheep, and horses. Furthermore, the classification system in [34] utilizes an mmWave dual receiver to distinguish between humans and animals. This system uses feedback signal responses from targets with a dual-receiver mmWave radar, utilizing a neural network based on synthetic 2D tensor data to categorize human and animal features [34]. However, none of these studies have utilized point clouds from mmWave radars for the simultaneous detection and tracking of people and animals. Although mmWave radars commonly collect data in IQ format, the point cloud format is advantageous in terms of external radar processing. Processing IQ data demands a large communication bandwidth and high computing power [35]. Moreover, receiving data directly in the point cloud format allows for the application of advanced data processing techniques like clustering and filtering with enhanced efficiency and speed.

**Figure 1:**
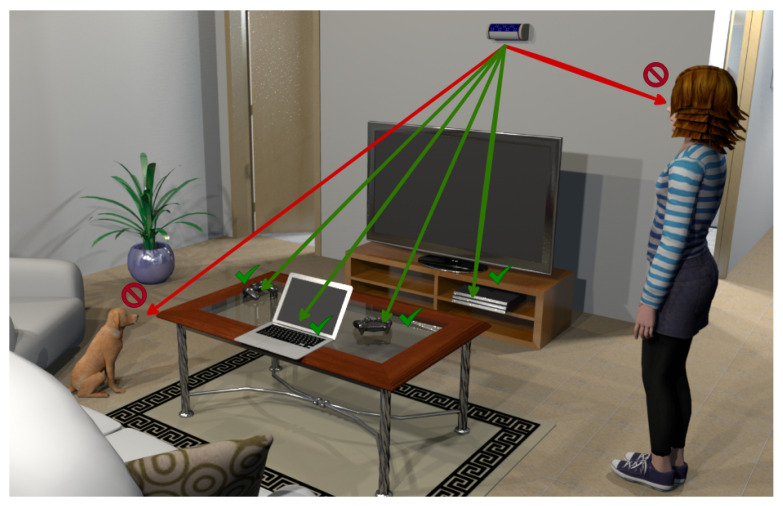
An illustrative scenario of the application of mmWave radars for safety-aware wireless energy transfer. A power beacon charges several devices. If the presence of humans or animals is detected by means of mmWave radars, potentially unsafe electromagnetic exposure can be avoided by turning off the power beacon or even by informing the power beacon to redesign the beams accordingly.

While some existing literature explores the use of multiple radars to detect people or objects, such as [16,18,30,31], and there are also studies that focus on animal classification, such as [33,34], none of the previous works address the simultaneous detection and tracking of people and small animals, such as dogs and cats, using multiple radar systems. Note that a system optimized for detecting people may be very inefficient in detecting small animals. A relevant application of simultaneous detection of humans and small animals is in autonomous vehicles. Moreover, another essential application of human and small animal detection is in the domain of wireless power transfer [36], ensuring safety in settings that involve wireless charging for electronic devices located in areas with the frequent presence of animals, the latter being the case with a modern living room, as illustrated in Figure 1. The appeal of wireless power transfer lies in its various benefits [37]. Notably, the convenience of avoiding connectors during device charging contributes to its attractiveness [37]. Additionally, a contactless solution proves more reliable, sidestepping issues like corrosion, dust intrusion, and moisture exposure [37]. To address potential health risks associated with electromagnetic fields, the wireless charging system can be intelligently deactivated upon detecting the presence of humans or animals in the environment [36].

### 1.2. Novelty and Contribution

This paper introduces a strategy for the simultaneous detection of humans and small animals employing multiple mmWave radars. The decision to limit our study to these two targets is with the intention of exploring one of the challenging scenarios for radar detection, particularly when potential targets significantly differ in terms of energy signatures, which depend on their size. It is considerably challenging to optimize a radar system to effectively detect targets with large deviations in energy signatures. If the radar setting is optimized to minimize false negatives for a target with a small energy signature, such as a small animal, this optimization could compromise the accuracy of detecting larger targets, like humans, thereby increasing the risk of false positives for the latter. Detecting multiple humans is in principle less challenging than detecting a small animal and a human in the same scene. Thus, our selection of a small animal and a human as targets stems from viewing them as an ideal benchmark for testing the limits of radar sensitivity and detection capabilities. Then, the primary objective is to demonstrate the enhanced detection efficacy achievable by multiple radars. It is shown that algorithms relying solely on a single radar may not capture sufficient reflection points from small animals, potentially leading to their misclassification as noise or remaining undetected. To the best of the authors’ knowledge, this is the first work to use point clouds from up to four mmWave radars to detect and track people and small animals in the same environment. We explore two different radar positioning scenarios and present a comparative analysis of their respective results. Furthermore, this study includes an examination of three data-fusion methodologies. Importantly, our focus is on target detection, not on the classification of the target. The goal is to highlight the increased efficacy in target detection using multiple radars, showcasing how this approach overcomes limitations associated with the use of a single radar for targets with diverse shapes and sizes.

The proposed system achieves 97.1% sensitivity and up to 91.4% precision in the detection of humans and small animals in an indoor environment, considering the best fusion strategy. The contribution of this article can be summarized as follows.

- We investigate the use of multiple mmWave radars to detect people and small animals, analyzing the impact of different data fusion and radar position strategies.
- We show that data fusion from multiple radars can significantly improve sensitivity and precision, enabling the simultaneous detection of small animals and humans.

The rest of this paper is structured as follows. The principles of mmWave radar are reviewed in Section 2. Section 3 describes the proposed approach, while Section 4 introduces the implementation details and the test setup. Section 5 evaluates the system, while Section 6 concludes the paper.

## 2. mmWave Radar Preliminaries

Radar systems emit electromagnetic waves that interact with objects in their path. By capturing reflected signals, these systems extract valuable information about the range, Doppler velocity, and angular positioning of the objects. Radars can be categorized into two types based on the signal they employ: frequency-modulated continuous wave (FMCW) radar and pulsed radar [15]. The radar used in this study, the IWR6843 industrial starter kit (ISK) 2.0 from TI, is an FMCW radar operating in the mmWave 60 GHz to 64 GHz band, equipped with four reception channels and three transmission channels [17].

**Figure 2:**
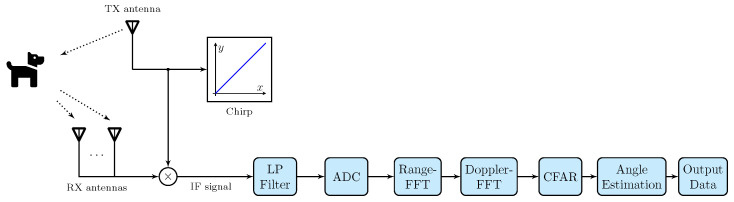
Diagram of the detection process of a target using an FMCW radar.

In the case of the FMCW radar, the transmitted signal is called a chirp, which is a sinusoidal signal characterized by a linear increase in frequency over time [15]. A chirp is characterized by initial frequency $f_{c}$, bandwidth *B*, and duration $T_{c}$. The slope *S* of the chirp defines the rate at which the frequency increases with time. A sequence of chirps forms a frame [15]. The illustration in Figure 2 presents the block diagram that describes the operational principle of an FMCW radar: a chirp is generated by a synthesizer, sent through the transmit (TX) antenna, and partially reflected by a target, and it finally reaches a set of receive (RX) antennas [15]. After mixing and low-pass filtering, an object in front of the radar generates an IF signal with a constant frequency [15]. Then, such an IF signal is sampled by an analog-to-digital converter (ADC), so that the ADC data are processed [15]. In the processor, the standard mmWave radar processing chain initially accepts ADC data as input. It then executes range and Doppler fast Fourier transform (FFT) operations, subsequently engaging in non-coherent detection through the implementation of the constant false alarm rate (CFAR) algorithm [38]. The final step involves estimating the angle using a 3D-FFT technique, which results in the generation of detected points termed point cloud data [38].

Moreover, the constant false alarm rate (CFAR) algorithm is one of the key technologies of radar signal processing [39]. It estimates the average power of the background according to the reference cells around the cell under test (CUT) as the threshold to detect targets, which maximizes the target’s detection probability while maintaining a constant probability of false alarm.

### 2.1. Output Data

The main information in the payload output by the radar is the point clouds, which contain reflections from the radar, positioning on the (x,y,z) axes, velocity, and signal strength. The term “radar point cloud” universally defines a compilation of detected objects reflected by the radar processing chain [40]. Originally, the concept of a point cloud emerged to characterize multi-dimensional data points derived from sensors like LIDAR and range cameras [40]. In some studies, point cloud data are described as a flexible information model commonly used to condense object signatures [40]. Essentially, point cloud data comprises numerous sets of individual points positioned uniquely in Euclidean space [40].

A primary advantage of this representation lies in its ability to convey crucial object information while demanding minimal computational and memory resources [40]. This quality renders it suitable for devices with limited resources, such as the TI mmWave radar [40]. Additionally, point clouds represent target signatures in point form, enabling the representation of complex targets using only a few data points. In contrast, a typical LIDAR point cloud data frame, sampled from scene surfaces, may contain thousands or millions of data points. This quantity surpasses the data points collected from a scene via mmWave radar, where streaming raw IQ data without additional hardware is impossible due to memory and hardware constraints in the single-chip radar [40].

### 2.2. Radar Configuration

The mmWave radar used, IWR6843ISK [17], provides a high degree of flexibility in the configuration of chirp parameters and also allows multiple chirp configurations within a single frame [38]. Among the many configurable parameters are the maximum and minimum detection distances of a radar sensor, range resolution (the ability to distinguish nearby objects), and parameters for maximum velocity, velocity resolution, and angular resolution. The threshold of the CFAR algorithm is also configurable, making it possible to filter out detected points outside the specified limits in the range domain or the Doppler domain. Initially, the configuration file is transmitted to the radar via a serial port, which requires a connection to a central processor and consumes a short period of time. However, once established, the configuration can be hard-coded, allowing the device to autonomously boot, configure, and emit chirps and transmit output data through a serial port without additional user intervention.

## 3. Proposed Approach

This work considers the use of point clouds generated by *M* different mmWave radars to detect both people and small animals. The proposed approach consists of three sequential modules: data acquisition, data fusion, and tracking.

(1) **Data Acquisition.**
  Each FMCW radar transmits mmWave chirps and records reflections from the scene. Subsequently, it processes the dynamic point clouds, identifying and eliminating points corresponding to static objects.
(2) **Data Fusion.**
  The data obtained by each of the radars are transformed into a common coordinate system so that a method for data fusion and clustering can be implemented.
(3) **Tracking.**
  The system associates the same human/small animal in consecutive frames and uses a multiple-object tracking algorithm to maintain their trajectories.

### 3.1. Data Acquisition

As previously stated, the FMCW radar operates by transmitting mmWave signals and capturing their reflections within a scene at a moment in time. The returned signal undergoes preliminary processing on the sensor, which then computes the point clouds. Reflections from static elements such as the ground, door frame, ceiling, walls, and furniture introduce a notable challenge [41]. To enhance the distinction between objects of interest and the background scene, a calibration step is incorporated into the system. In the installation phase, the device captures radar returns from the background, establishing a reference dataset. This recorded background information is then subtracted from the current frame during operation, facilitating the identification of newly introduced objects in the scene [41].

The resulting data are transmitted into a central processor, where rotation and translation matrices are computed individually for each radar, incorporating their specific orientations and positions within the system. This process is facilitated by the known spatial coordinates and orientations of each radar unit. Subsequently, the data acquired from each radar undergo a transformation to align with a unified coordinate system, ensuring a consistent and coherent spatial reference across all radar sources.

### 3.2. Data Fusion

The generated points of each radar are placed into one coordinate system, and the data go through a clustering process. Three data-fusion methodologies are evaluated.

#### 3.2.1. Method 1 [18]—Intersection of Detected Data

**Figure 3:**
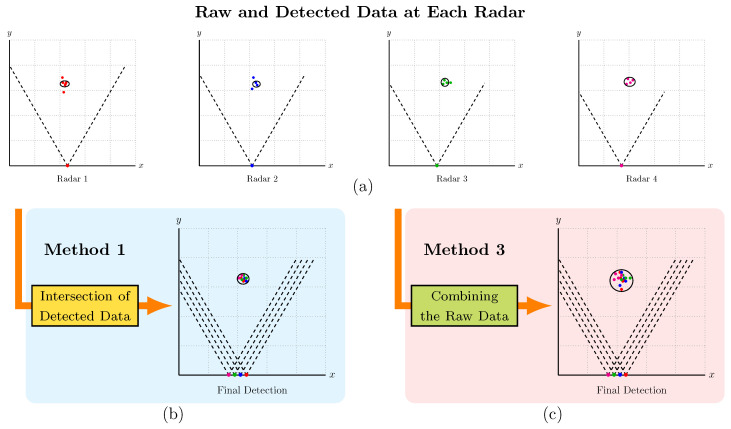
The raw and detected data at each radar are shown in (**a**). Method 1, based on the intersection of individually detected data, is illustrated in (**b**). Method 3, based on the combination of raw data, is shown in (**c**).

The first approach is based on the method introduced in [18] and is illustrated in Figure 3, considering M=4 radars. In Figure 3a, we present the raw data from each radar, as points in different colors. The data gathered by each radar, stored in (x,y,z) coordinate formats, is processed via the density-based spatial clustering of applications with noise algorithm (DBSCAN). In the realm of density-based clustering algorithms, DBSCAN stands out as a widely embraced algorithm within this classification [21], having demonstrated successful application in clustering radar point clouds, as indicated in [7,16,18,21]. A major feature is that it does not require the number of clusters to be specified a priori [7]. Furthermore, DBSCAN detects clusters of arbitrary shapes, while it can automatically mark outliers to cope with noise, enhancing its effectiveness in handling noisy data [7,42].

The assignment of a point to a cluster in the DBSCAN algorithm depends on the neighborhood of the point around a radius ϵ [42]. Then, this algorithm classifies points into three distinct categories: core, a point within a cluster that boasts a minimum of *minpts* neighbors within its ϵ-neighborhood; border, a point within a cluster that possesses fewer than *minpts* neighbors in its ϵ-neighborhood; and noise, an outlier that does not align with any cluster [42].

The clusters detected by each radar are illustrated by ellipses in Figure 3a. After evaluating the clusters’ dimensions and positions, the system proceeds to compute the eigenvectors specific to each cluster [18]. Subsequently, the algorithm estimates distances and identifies overlapping regions between clusters from different radars, preserving the groups where the centroids align closely and most of their areas intersect [18]. Unlike the methodology in [18], which uses only two radars, here, we extend the method for up to four radars. Consequently, in this case, a positive decision necessitates detection from all radars; otherwise, the input is classified as noise. Figure 3b illustrates the final result of this method by another ellipse. Note that all radars must detect the target; otherwise, it is not detected in the final step. This can be a problem for detecting small animals, as they generate fewer points than humans and may not be detected by all radars simultaneously, thus potentially missing detection. Thus, one should expect a decrease in sensitivity with the increase of *M*. This issue can be alleviated by considering a relatively small value of *minpts* in DBSCAN, but at the potential cost of increasing the occurrence of false positives.

#### 3.2.2. Method 2—*R* out of *M*

The second fusion method is a modified version of Method 1 [18]. Unlike the original methodology, where detection relied on the intersection of individual detections of all *M* radars, this adapted method introduces flexibility by varying *R*, the minimum number of detecting radars to confirm a detection event. The approach of Method 1 is applied in each possible combination of *R* out of *M* radars, leading to M!/(R!(M−R)!) possible intersections. For instance, in the case of M=4 radars and R=2, the method proposed in [18] is applied separately in each possible pair among the four radars. Taking Figure 3a as an example, Method 2 would consider the following set of intersections: {(Radar 1, Radar 2), (Radar 1, Radar 3), (Radar 1, Radar 4),(Radar 2, Radar 3), (Radar 2, Radar 4), (Radar 3, Radar 4)}. In this example, it is sufficient for a target to be successfully detected if any R=2 of the M=4 radars detect it. Clearly, when R<M, the sensitivity should be increased with respect to Method 1, but at the cost of precision.

#### 3.2.3. Method 3—Combining the Raw Data

In the third and final method, clustering is not applied individually in the raw data of each radar, as in Methods 1 and 2 above. Rather than using individual radar data independently, the collected data from all radars undergo processing in a unified coordinate system through the DBSCAN algorithm. Consequently, the point clouds from each radar are collectively considered for clustering. The procedure is illustrated again with the aid of Figure 3, where the final result of Method 3 is shown in Figure 3c. Therefore, unlike Method 1, when the number of radars *M* increases, the sensitivity also tends to increase due to the availability of more points, making undetected targets much less frequent.

### 3.3. Tracking

**Figure 4:**
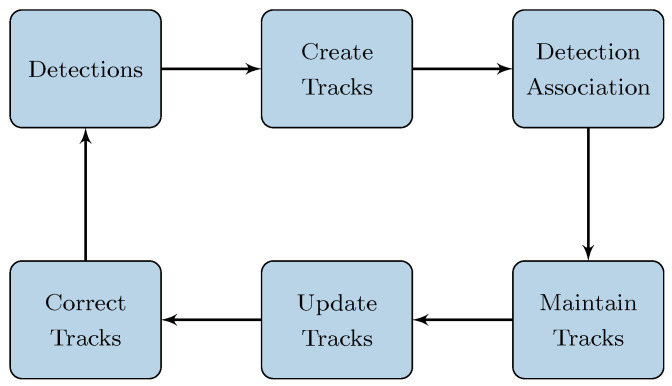
Block diagram of the proposed tracking process.

To enhance the detection rate, a tracking algorithm is implemented, similar to the one proposed in [7]. The tracking module takes as input a vector of cluster measurements, including positioning on the (x,y,z) axes and velocity information from the radars. Tracking both a human and a small animal through the continuous capture of individual point clouds requires the efficient temporal association of detection, alongside noise correction and prediction in sensor data. The flow of the multi-target tracker system is illustrated in Figure 4.

In this work, tracks are established to detect multiple individuals, whether people or small animals, in each frame. While [7] utilizes the Hungarian algorithm for target association across frames, this work opts for James Munkres’s variant of the Hungarian assignment algorithm [43]. The Hungarian algorithm represents a combinatorial optimization method [7]. It operates through a distance matrix, which holds the Euclidean distances between every pair of tracks along the matrix rows and detections in the columns [44]. These distances are computed from the centroids of predicted and detected objects, where smaller distances correspond to a greater likelihood of correctly associating detections with predictions [44].

The main difference between the Hungarian algorithm and the Munkres variant lies in how it iterates through the cost matrix to find the optimal solution for assignment problems. The Munkres algorithm employs alternating path and labeling techniques to identify and update assignments more efficiently, reducing computational complexity compared to the original version of the Hungarian algorithm [43]. Similar to [7], a new track is initiated for each detection, originating either from the first incoming frame or those not associated with an existing track. Tracks that remain undetected for a continuous duration of *U* frames are flagged as inactive and excluded from subsequent associations. Furthermore, a Kalman filter is employed for trajectory forecasting and adjustments. Further elaboration on these processes is provided below.

#### 3.3.1. Tracks Creation and Association

At the beginning of the tracking process, an empty track is created, with each track being a structured representation of a target detected by the radars. This structured format aims to maintain the state of a tracked target. After data fusion, centroids and bounding boxes are returned if any target is detected. To maintain continuous tracking of individual point clouds for people or animals, an effective temporal association of detection is crucial.

The association method assigning detections tracks is facilitated through the application of James Munkres’s variant of the Hungarian algorithm, which manages the assignment problem between existing tracks and new detections [45]. The association process employs a cost matrix **C**, with rows representing tracks and columns representing detections [43]. The element $C_{ij}$ in the matrix delineates the cost of assigning detection *j* to track *i* [43], and it is calculated using the Euclidean distance between the predicted location of the track and the detected object’s centroid:

(1) $$C_{ij}=\sqrt{{\left(x_{\mathrm{track},i}-x_{\mathrm{detect},j}\right)}^{2}+{\left(y_{\mathrm{track},i}-y_{\mathrm{detect},j}\right)}^{2}},$$

where $x_{\mathrm{track},i}$ and $y_{\mathrm{track},i}$ are the coordinates of the *i*-th track’s predicted position, and $x_{\mathrm{detect},j}$ and $y_{\mathrm{detect},j}$ are the coordinates of the *j*-th detection. The algorithm then processes this cost matrix to determine the optimal assignment of detections to tracks, minimizing the total cost [45]. This yields the indices for both assigned and unassigned tracks and detections, allowing for the updating of existing tracks and the creation of new ones for unassigned detections. Through this method, the tracking system ensures the continuous monitoring of targets by dynamically managing the creation of new tracks and the association of detections to existing tracks, optimizing the tracking process over time [45].

#### 3.3.2. Track Prediction, Update and Correction

To effectively track an object’s movement across frames, predicting its future location is important. The previous motion patterns feed the predictions [45], which are performed using a Kalman filter. The Kalman filter is essential for predicting an object’s future location, accounting for process noise (**Q**) and measurement noise (**R**). It maintains a state (**x**) for each track, comprising location and velocity along the (x,y,z) axes. The state for each track at time *k* is updated based on the previous state at time k−1 and the current measurements [46,47]. The state prediction equation is given by

(2) $${\hat{x}}_{k|k-1}=F_{k}x_{k-1}+B_{k}u_{k},$$

where ${\hat{x}}_{k|k-1}$ is the predicted state estimate at time *k*, given all available information up to time k−1, $F_{k}$ is the state transition model applied to the previous state $x_{k-1}$, $B_{k}$ is the control input model applied to the control vector $u_{k}$, which represents any known external influences on the state, while $x_{k-1}$ is the state estimate at time k−1 [46].

The covariance prediction equation is

(3) $$P_{k|k-1}=F_{k}P_{k-1}F_{k}^{⊤}+Q_{k},$$

where $P_{k|k-1}$ is the predicted estimate covariance [46].

In the tracking algorithm, the function responsible for updating each assigned track seamlessly incorporates the corresponding detection information. It accurately calls the method to correct the location estimate, storing the new bounding box in the process. This update is performed in a frame-by-frame manner during the post-processing stage. When a new measurement $z_{k}$ is received, the update and correction steps are performed as follows [46]:

(4) $$\begin{array}{cc} K_{k} & =P_{k|k-1}H_{k}^{⊤}{\left(H_{k}P_{k|k-1}H_{k}^{⊤}+R_{k}\right)}^{-1} \end{array}$$

(5) $$\begin{array}{cc} x_{k} & ={\hat{x}}_{k|k-1}+K_{k}\left(z_{k}-H_{k}{\hat{x}}_{k|k-1}\right) \end{array}$$

(6) $$\begin{array}{cc} P_{k} & =\left(I-K_{k}H_{k}\right)P_{k|k-1} \end{array}$$

where $K_{k}$ is the Kalman gain, $z_{k}$ is the measurement vector, and $H_{k}$ is the measurement model. In addition, $P_{k}$ is the updated estimate covariance, and *I* is the identity matrix.

These steps ensure that the tracker accurately predicts the object’s movement across frames, incorporating both model predictions and real measurements to refine the position and velocity estimates [45].

#### 3.3.3. Track Maintenance

Within each frame, detections are either linked to existing tracks or remain unlinked, leading to what we term “invisible” tracks for those without corresponding detections. New tracks are initiated from unassigned detections. Importantly, we manage each track’s visibility by incrementally tracking the number of consecutive frames it remains unlinked. This count is crucial for determining when a track should be considered inactive and subsequently removed, indicating that the object has likely exited the observable area.

For a given track $T_{i}$, let us denote its visibility count as $V_{i}$, a method to avoid confusion with the cost matrix *C*. This visibility count is updated as follows:

(7) $$V_{i}=\left\{\begin{array}{cc} 0 & \mathrm{if}\;T_{i}\;\mathrm{is}\;\mathrm{linked}\;\mathrm{to}\;a\;\mathrm{detection},\\ V_{i}+1 & \mathrm{if}\;T_{i}\;\mathrm{is}\;\mathrm{not}\;\mathrm{linked}\;\mathrm{to}\;a\;\mathrm{detection}. \end{array}\right.$$

A track is considered for removal if its visibility count $V_{i}$ exceeds a predefined threshold θ, indicating prolonged absence from the field of view:

(8) $$\mathrm{if}\;V_{i}>\theta ,\;\mathrm{then}\;\mathrm{remove}\;T_{i}.$$

This mechanism underlines the dynamic nature of tracking, where the sensitivity and accuracy are notably enhanced by the process [45].

**Figure 5:**
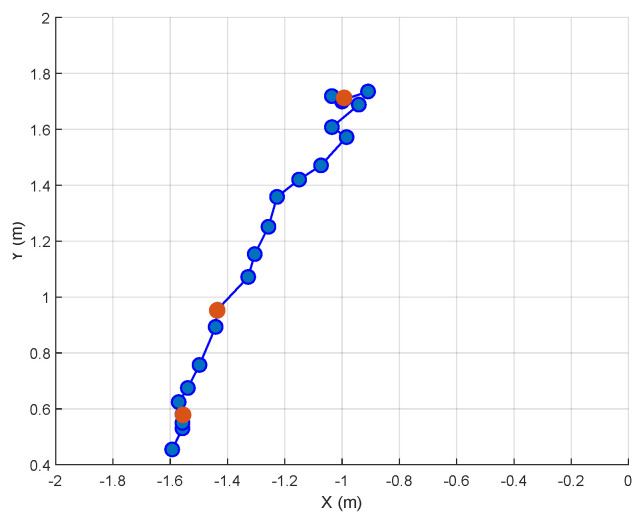
Two-dimensional superior view plot example of an animal being tracked using the tracking algorithm. One small animal was present in the scene, and four radars were utilized. The blue dots are the radars’ detections that were confirmed by the tracking, while the red dots are those detections missed by the radars but that were included by the tracking process.

Figure 5 displays the trajectory of an identified target. In this scenario, a small animal enters the scene and moves toward the positive *y*-axis. Four radars are employed for detection. The blue dots in Figure 5 represent the detections made by the radars that were confirmed by the tracking process. The red dots are the detections that were missed by the radars but that were included after the tracking process. Note the relevance of tracking in this application, as it clearly increases the sensitivity.

## 4. Implementation

### 4.1. Radar Configuration

As the purpose of this work is to detect not only humans but also small animals, the typical configurations provided by the manufacturer need to be adjusted to fit the project goals. An adequate threshold was set for the CFAR algorithm with the aim of obtaining sufficient data for post-processing. Given the objective to detect both small animals and humans, capturing a greater number of point clouds than those solely for humans is crucial, as animals may generate fewer point clouds due to their smaller size and distinct shapes. The radars are configured for an indoor environment, with a maximum distance of 5 m, a range resolution of 7 cm, a maximum radial velocity of 2.4 m/s, a velocity resolution of 0.15 m/s, and a frame duration of 100 ms. The mmWave radars were configured for an azimuth opening angle of 120° and an elevation angle of 30°.

When employing multiple radars, understanding signal interference becomes crucial. As stated in [18], the probability of interference remains below 1% when utilizing four radars, but this probability escalates with the use of more than ten radars. In such cases, explicit synchronization among the radars or the implementation of an interference detection algorithm becomes necessary. Consequently, it can be inferred that the likelihood of interference is minimal when concurrently operating up to four radars, aligning with the approach proposed in this study.

### 4.2. Setup

The experiments were conducted in a 4 m × 4 m room. The animal detected in the test was a small dog, weighing approximately 3 kg and 40 cm tall. During tests, a camera was used to monitor the environment, and the radars operated in an unsynchronized manner. The camera acted as an auxiliary means of observation to validate the presence or absence of targets within the environment. Therefore, its role was important in the sense of corroborating the radar’s detection capabilities. By comparing the visual evidence captured by the camera with the radar’s detection outcomes, we could assess the accuracy and reliability of our radar system more effectively. As detecting a small animal is more challenging than detecting a human, we set it that during 66.67% of the time, only the animal was in the area, and 33.33% of the time, there was a human and an animal. The system was operated for 3000 frames.

**Figure 6:**
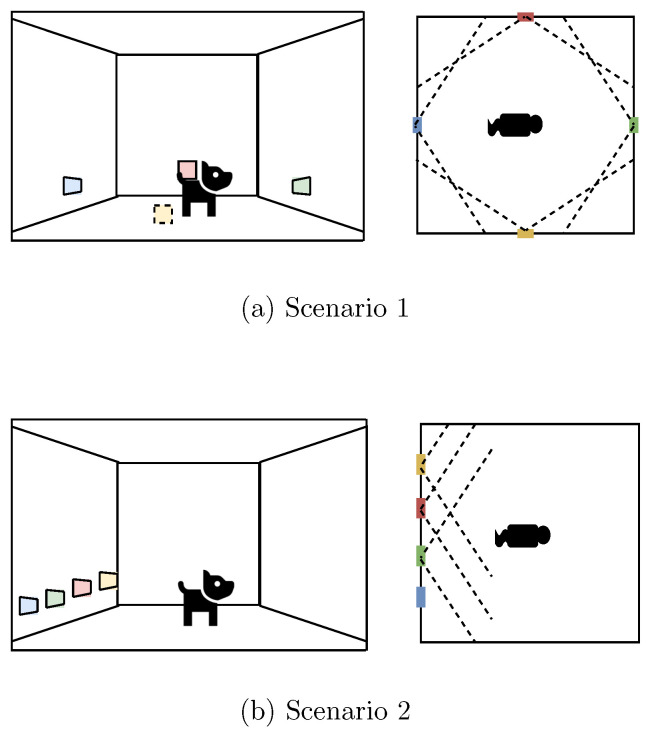
The two scenarios with their radar placement and field of view for human and animal detection tests.

Once configured, the radars were placed in two different scenarios for the tests. In the first scenario, the radars were horizontally aligned, as shown in Figure 6a, separated by the same distance between them. In the second scenario, the radars were each positioned close to one of the four walls, at the center of each wall, all pointing towards the center of the room, as depicted in Figure 6b. These two scenarios were proposed to analyze the detection performance in different radar positions and to obtain data at different angles. In the images, the dashed line illustrates the opening angle of the radars. Given the radar’s lower elevation angle compared to the azimuth angle as a result of antenna disposition, the radars were placed at the typical animal’s height. Following the manufacturer’s suggestions, none of the radars were placed on the ceiling [48]. These placement variations were intended to explore different perspectives and angles for data collection, providing a comprehensive analysis of the system performance in various configurations. In our setup, each radar was positioned to ensure there were no obstructions directly in front of it, facilitating unimpeded detection capabilities. Moreover, careful consideration was given to positioning the radars in locations where targets are most likely to be detected within their field of view while minimizing the presence of obstructive elements. Despite the potential for challenges posed by physical obstructions, the advanced nature of current radar systems enables the detection of targets even through certain barriers, such as glass surfaces. This capability significantly increases the flexibility and effectiveness of radar systems in complex environments. However, optimizing radar placement transcends simply overcoming physical obstructions; it also entails strategic positioning to maximize the field of view of the radar array, ensuring comprehensive coverage and enhanced detection accuracy. This strategic approach to radar placement, combined with a calibration technique, provides a robust solution to the challenges of data fusion in practical applications.

### 4.3. DBSCAN Algorithm

For each one of the data fusion methods and for each radar placement, the DBSCAN algorithm was calibrated to detect both people and small animals. The parameters of DBSCAN mentioned in Section 3.2.1 were adjusted to improve target detection and minimize the creation of false targets. Small animals typically generate fewer reflection points, even with a lower threshold in the CFAR algorithm, while humans tend to produce more reflection points, posing a challenge for simultaneous detection.

The adjustments in DBSCAN parameters were made to address this challenge and optimize the algorithm’s performance. The goal was to enhance target detection while avoiding the generation of excessive false positives. It is worth noting that the use of multiple radars contributes to generating a sufficient number of points, improving the overall effectiveness of the detection system, especially for small animals.

### 4.4. Tracking Algorithm

The tracking algorithm was implemented to improve the detection rate and prevent the generation of ghost targets. The algorithm thresholds are configured with the aim of improving the detection rate, especially when using fewer radars. It is important to note that depending on the distance and movement of the target, there might be frames where no point cloud data are transmitted, emphasizing the importance of tracking for successful detection.

### 4.5. Performance Metrics

The evaluation of the detection system involved the use of the following key metrics (The F1-score of each test was also analyzed; however, it did not yield different conclusions from those shown by precision and sensitivity. Therefore, for the sake of brevity, it is omitted here):

- **Positives**
  (P): Human and/or animal present in the area.
- **True Positives**
  (TP): Human and/or animal in the detection area that is successfully detected by the radar.
- **False Positives**
  (FP): Noise or other objects in the detection area that are falsely detected as humans or animals.
- **Sensitivity**
  (TP/P): The ability to detect humans and/or animals when they are in the detection area.
- **Precision**
  (TP/(TP + FP)): The ability to distinguish human and/or animal from false detection.

An ideal system should exhibit high sensitivity and high precision [18], but that is a very challenging task. Moreover, in safety-related applications, such as those related to wireless energy transfer [36], sensitivity is more relevant than precision.

## 5. Results

Tests were carried out using the two radar-placement options mentioned in Section 4, and the three data fusion methods in Section 3.2 were applied.

### 5.1. Single Radar

First, a test was performed using a single radar in order to highlight the motivation to use multiple radars. For the sake of brevity, the results are presented only for the first scenario, where the radars are positioned side by side and utilize the tracking algorithm. The conclusions are very similar for the second scenario. Then, the sensitivity and precision achieved are presented in Table 1. The parameters used for human detection were those proposed in [7], while for the detection of small animals, the number of required point clouds was reduced to around 1/8 of the total points. In the optimized scenario for detecting both humans and small animals, the parameters were fine-tuned to achieve optimal performance, aiming at high sensitivity with balanced precision, avoiding significant discrepancies between the two parameters.

**Figure 7:**
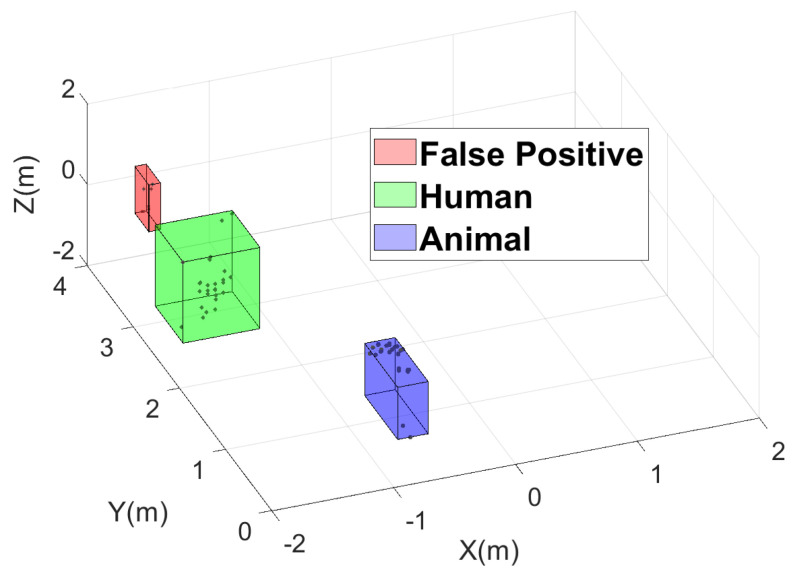
Detection of three targets in a scene that contained only two (a small animal and a human) using optimized DBSCAN parameters for the detection of small animals only.

Analysis of the data in Table 1 reveals notable differences: when using DBSCAN parameters specifically optimized for the detection of small animals, higher sensitivity is achieved, as expected, but a larger incidence of ghost detections is also observed, reducing precision. This situation is illustrated in Figure 7, which shows the results of the DBSCAN algorithm in a situation where both an animal and a human were present in the scene. Note that an additional ghost target was detected. In contrast, when optimizing the parameters for human detection, there is a decrease in true positives, often resulting in the failure to detect the animal and a decrease in sensitivity, leading to the results shown in Table 1, where the sensitivity is severely compromised, but the precision becomes very high. Finally, when the system is optimized to detect both humans and animals, a more balanced performance is achieved, but it is probably still insufficient for many applications, such as those related to safety. A possible solution to increase both the sensitivity and the precision is to use multiple radars.

**Figure 8:**
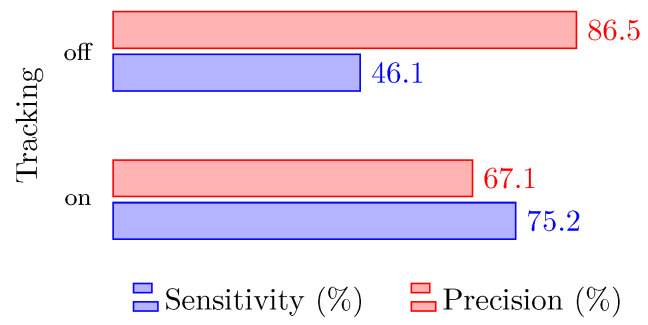
System performance with and without the tracking algorithm for Scenario 1 and a single radar.

Another test was conducted switching the tracking algorithm on and off. The test considered the optimized DBSCAN parameters for detecting both humans and animals, and the results are shown in Figure 8. Examining the data makes it evident that the integration of the tracking algorithm significantly increases the sensitivity, which is fundamental for high-performance applications. In the next subsection, the performance of the tracking algorithm with multiple radars is presented.

### 5.2. Multiple Radars

**Figure 9:**
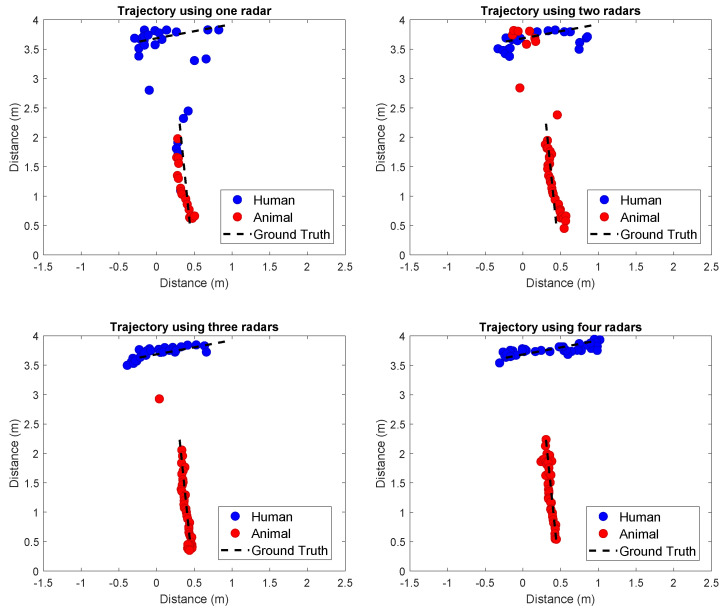
Operation of the tracking algorithm with 1 to 4 radars. Each subfigure illustrates the tracking behavior as the number of radars increases.

Next, we discuss the results of applying the methodology proposed in Section 3, considering the three data-fusion strategies and the two radar-placement scenarios. First, we demonstrate the algorithm performance in tracking a person and an animal, aiming to discern the algorithm behavior with the employment of multiple radar systems. Specifically, in this case, we utilized the third fusion method. The results obtained are displayed in Figure 9. It becomes clear that tracking is enhanced with the use of multiple radars; the analysis reveals that while a single radar setup provides a baseline capability for object tracking, the integration of two, three, or four radars significantly amplifies the sensitivity and accuracy. Notably, it is observed that when employing one and two radars, the system occasionally confuses the tracks, mistakenly swapping the person for the animal and vice versa. This issue, however, is effectively mitigated with the deployment of three and four radar configurations, wherein such inaccuracies do not occur. This progressive enhancement in tracking performance underlines the importance of multi-radar configurations for high-fidelity tracking in complex environments.

The progressive enhancement in tracking performance underlines the importance of multi-radar configurations for high-fidelity tracking in complex environments. It is important to underscore a key advantage of our multi-radar configuration, which is particularly demonstrated in scenarios of visual obstruction, such as when a large human obscures a small animal from the view of one radar. In these cases, the probability remains high that other radars in the system will have an unobstructed view of the animal, ensuring its continuous detection and tracking. This benefit is notably pronounced in our implementation of the second and third fusion methods, where the detection of a target by all radars is not a prerequisite for its positive detection. Such a feature underscores the strategic advantage of employing multiple radars, as it allows for the maintenance of tracking accuracy and system resilience even when individual radars face visual obstructions.

**Figure 10:**
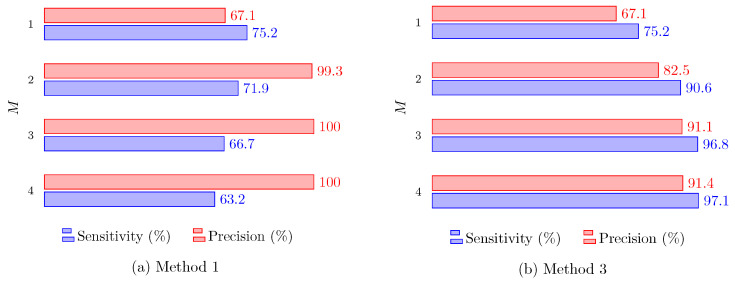
Precision and sensitivity for Methods 1 and 3 in the first scenario versus the number of radars *M*.

Figure 10 shows the precision and sensitivity results for data fusion Methods 1 and 3, respectively, “Intersection of Detected Data” and “Combining the Raw Data”, versus the number of radars *M*. Clearly, when the number of radars increases, Method 1 performs better in terms of precision but loses considerably in terms of sensitivity. This is because every radar must detect the target to be finally considered detected. In the case of small animal detection, it is plausible that radars might not simultaneously detect the target, reducing the sensitivity. With the same arguments, when all radars detect a target, it is very probable that this is a true positive, increasing the precision.

**Figure 11:**
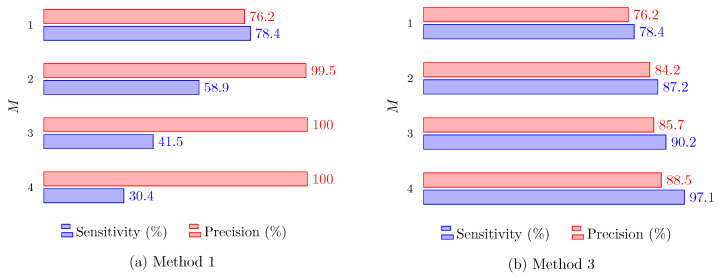
Precision and sensitivity for Methods 1 and 3 in the second scenario versus the number of radars *M*.

Note that a completely different behavior is observed with Method 3, as both precision and sensitivity increase with *M*. As this method includes all available raw data in a unique clustering process, having more radars improves performance in both aspects, achieving more than 90% in sensitivity and precision for M≥3. Such a capability to achieve high sensitivity and high precision at the same time is very interesting from the point of view of safety-related applications. Figure 11 shows similar results, but for the second scenario, where the radars are on each of the walls. The same trends are observable, although it is clear that a better performance was obtained in the first scenario, where the radars are side by side.

**Figure 12:**
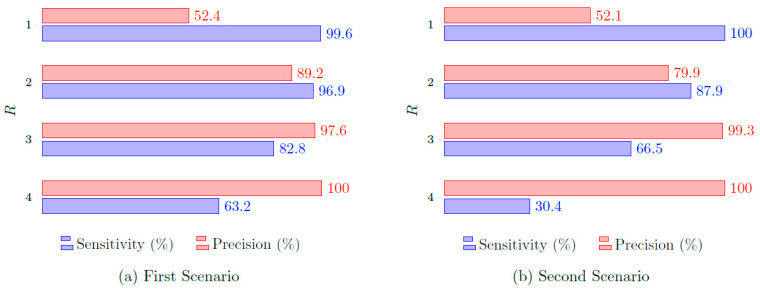
Precision and sensitivity for Method 2 in the first and second scenarios, considering M=4 radars, versus *R*, the number of radars required for detection.

Figure 12 illustrates the performance of Method 2 for both scenarios. Recall that Method 2, *R* out of *M*, is an alternative to Method 1, where here only *R* of the *M* radars have to detect a target to be finally detected. We consider M=4 radars and vary *R* from 1 to 4. Note that a much more balanced performance than that obtained by Method 1 can be achieved, especially with R=2 and for the first scenario. That is very reasonable since a positive detection can now be achieved even if some of the radars missed the target. However, as illustrated in Table 2, where we consider only the best-performing configurations for each method, the performance of Method 3 is still the best, being able to achieve both higher sensitivity and higher precision than Method 2.

## 6. Conclusions

In this work, the detection and tracking of humans and small animals was investigated using multiple mmWave radars. First, the sensitivity of using a single radar to detect humans and animals simultaneously was shown to be relatively low, motivating the use of multiple radars. Then, two radar-positioning scenarios and three data-fusion strategies were analyzed. We showed that the data-fusion strategy that combines the raw data before applying a clustering algorithm performs best, achieving high levels of sensitivity and precision. The results demonstrate that the use of multiple radars to detect people and small animals is very promising, even in safety-related applications where sensitivity must be high. A somewhat straightforward extension of this work would be the application of multiple radars to detect both humans and animals in outdoor environments, as well as in settings with animals and people of different sizes. A perhaps more challenging and rewarding future work would be the fusion of radar data with other technologies so that high sensitivity and precision can be achieved with fewer sensors.

Moreover, despite the success in achieving high sensitivity in the detection and tracking of humans and animals, we may encounter limitations when the targets remain in close proximity over extended periods, moving together. Thus, another potential future work is the thorough investigation of the effects, and the corresponding solutions, of grouped targets on tracking accuracy.

Finally, as a practical step forward, we plan the construction of a prototype system for wireless power transfer informed by multiple radars for the detection of both people and animals. This system holds the potential to enhance safety and efficiency in various environments, addressing the unique challenges posed by the coexistence of humans and animals of different sizes.

## Figures and Tables

**Table 1:** System performance with DBSCAN thresholds optimized for animal detection only, for human detection only, or for both, with a single radar in the first scenario.

DBSCAN Optimized for	Precision	Sensitivity
Small animals	52.8%	81.4%
Humans	100%	32.6%
Both	67.1%	75.2%

**Table 2:** Best performance for Methods 2 and 3 in Scenarios 1 and 2.

	Scenario 1		Scenario 2	
**Method**	**Precision**	**Sensitivity**	**Precision**	**Sensitivity**
Method 2	89.2%	96.9%	79.9%	87.9%
Method 3	91.4%	97.1%	88.5%	97.1%

## Data Availability

Data are contained within the article.

## Abbreviations

The following abbreviations are used in this manuscript:

ADC	Analog-to-Digital Converter
AoA	Angle of Arrival
CFAR	Constant False Alarm Rate
CUT	Cell Under Test
DBSCAN	Density-Based Spatial Clustering of Applications with Noise
FFT	Fast Fourier Transform
FMCW	Frequency Modulated Continuous Wave
FP	False Positive
IF	Intermediate Frequency
IoT	Internet of Things
IQ	In-Phase and Quadrature
LIDAR	Light Detection and Ranging
mmWave	Millimeter Wave
P	Positive
RX	Receiving Antenna
STF	Strong Tracking Filter
TI	Texas Instruments
TP	True Positive
TX	Transmitting Antenna
